# Timing of hip hemiarthroplasty and the influence on prosthetic joint infection

**DOI:** 10.1371/journal.pone.0229947

**Published:** 2020-03-12

**Authors:** Nolan S. Horner, Kirsten M. Grønhaug Larsen, Eleonor Svantesson, Kristian Samuelsson, Olufemi R. Ayeni, Jan-Erik Gjertsen, Bengt Östman

**Affiliations:** 1 Division of Orthopaedic Surgery, Department of Surgery, McMaster University, Hamilton, Ontario, Canada; 2 Department of Orthopaedic Surgery, Østfold Hospital Trust, Kalnes, Norway; 3 Department of Orthopaedics, The Sahlgrenska Academy, Institute of Clinical Sciences, University of Gothenburg, Gothenburg, Sweden; 4 Department of Clinical Medicine (K1), Faculty of Medicine and Dentistry, University of Bergen, Bergen, Norway; 5 Department of Orthopaedic Surgery, Norwegian Hip Fracture Register, Haukeland University Hospital, Bergen, Norway; University of Ulsan College of Medicine, Asan Medical Center, REPUBLIC OF KOREA

## Abstract

**Introduction:**

Previous research suggested that patients have increased risk of infection with increased time from presentation with a femoral neck fracture to treatment with a hip hemiarthroplasty (HHA). The purpose of this study was to determine if rates of prosthetic joint infections within 3 months of surgery was affected by the time from patient presentation with a femoral neck fracture to the time of treatment with HHA.

**Materials and methods:**

Acute hip fractures treated with HHA between 2005 and 2017 at three centres in Norway were enrolled in the study. Multi-trauma patients were excluded. Univariable analysis was performed to determine any significant effect of pre-operative waiting time on infection rate. Two pre-planned analyses dichotomizing pre-operative waiting time cut-offs were performed.

**Results:**

There were 2300 patients with an average age of 82 (range, 48–100) years included of which 3.4% experienced a prosthetic joint infection within 3 months. The primary analysis found no significant difference in infection rate depending on time to surgery (OR = 1.06 (95% CI 0.94–1.20, p = 0.33)). The secondary analyses showed no significant differences in infection rates when comparing pre-operative waiting time of <24 hours vs ≥24 hours (OR = 0.92 (95% CI 0.58–1.46, p = 0.73)) and <48 hours vs ≥48 hours (OR = 1.39 (95% CI 0.81–2.38, p = 0.23)).

**Conclusion:**

Based off of a large retrospective Norwegian database of hip fractures there did not appear to be a significant difference in infection rate based on pre-operative wait time to surgery.

## Introduction

Femoral neck fractures are frequently treated with either hip hemiarthroplasty (HHA) or total hip arthroplasty (THA) [1]. Deep prosthetic joint infections (PJI) represent one of the most devastating complications of hip arthroplasty as they are associated with a high rate of morbidity and mortality [2]_,_[3]. PJI are also economically costly to healthcare systems [4].

Compared with elective THAs, acute arthroplasties for hip fractures are known to have further increased rates of mortality associated with PJI [5]_,_[6]. The reported rates of PJI after hip arthroplasty for hip fractures varies dramatically within the available literature [5]_,_[7]_,_[8]. Systematic reviews have reported rates of PJI after hip arthroplasty for hip fracture to vary between 0–18% for individual studies, with an average PJI rate of 2–4% [9],[10],[11]. Many studies have reported that patient factors such as age, obesity, and smoking status have significant effects on the risk of PJI [12]_,_[13]. However, the literature investigating risk factors for PJI after hip arthroplasty has mostly focused on the elective patient population, as opposed to hip fracture patients. Evidence does seem to suggest that hip fracture patients are at higher risk for PJI after hip arthroplasty [6]. A study from the Nordic Arthroplasty Register Association showed that the highest risk of revision due to PJI after hip arthroplasty was within the first 3 months after the operation [14]. A previous study by Westberg *et al*. suggested that an increased time from presentation with an acute hip fracture to surgery resulted in an increased risk of PJI [15]. However, this study was limited by a relatively low sample size of 184 patients and only PJI that occurred within 4 weeks from treatment were included. More recently Zajonz *et al*. found no significant difference in rates of periprosthetic infections after HHA for femoral neck fractures based on pre-operative waiting time[16]. Therefore, a larger study examining this topic would be useful to further examine whether pre-operative waiting time influences rates of PJI in hip fracture patients treated with HHA.

The primary purpose of this study was to determine if the rate of PJI was affected by the time from patient presentation in the emergency department with a hip fracture to the time of HHA. As a secondary purpose, the study aimed to evaluate which patient factors differed amongst patients who developed a PJI and those who did not, as well as which variables correlated with increased pre-operative waiting time.

## Materials and methods

All patients at three centres in Norway (Østfold Hospital Trust–Fredrikstad, Kalnes, and Moss hospitals) who were living in Østfold county and were treated with HHA using a lateral approach for first presentation of an acute hip fracture between 2005 and 2017 were consecutively included in this retrospective study. Only patients with a minimum of 3 months follow-up were included. Multitrauma patients, pathologic fractures, cementless hemiarthroplasties and patients with additional fractures that had to be operated on prior to HHA were excluded. Patients with ongoing antibiotic treatment for a bacterial infection at the time of presentation were also excluded. The first of four doses of cephalotin (2g q6h) was administered intravenously within one hour of surgery and the remaining dosages were given post-operatively. In the case of allergy three doses of clindamycin (600mg q8h) were used.

The primary research question was to determine whether pre-operative waiting time had an influence on PJI rates within the first 3 months post-operatively. Pre-operative waiting time was defined as the time from patient presentation in the emergency department to time of surgery. PJI were clinically diagnosed according to the Centers for Disease Control definition of deep incisional surgical site infection [17]. Two superficial infections with positive cultures treated with local skin debridement and a brief period of antibiotics were also included in the PJI rate. All data which included demographic information, the presence and timing of PJI, type of procedures performed, culture results, type of hip fractures and duration of surgeries were obtained through electronic medical records and x-ray images. Using the unique identification number assigned for each resident in Norway, data extracted from the hospitals’ electronic medical records to a local register were validated against data exported from the Norwegian Hip Fracture Register (NHFR)[18]. The validation included the principal diagnosis, type of arthroplasty, date of primary operation, type of antibiotic prophylaxis, date and cause of reoperation, and laterality. In case of inconsistency, the hospitals medical records and digital pre- and post-operative x-rays were reviewed. The study was approved by the Regional Committee for Medical and Health Research Ethics Norway (approval no 2017/2453D).

### Statistical analysis

A univariable analysis was performed to determine if pre-operative waiting time significantly (p<0.05) altered PJI rate within 3 months after hip arthroplasty. A multivariable regression would also be performed if there was any demographics found between the PJI and non-PJI groups that were determined to be confounding variables. Two *a priori* criteria had to be met in order for a variable to be considered a confounder and therefore included in a multivariable analysis: 1) the variable was different (p<0.10) between the group of patients who sustained a PJI within 3 months and those who did not; 2) the variable had a correlation (p<0.10) with pre-operative waiting time. Univariable logistic regression was used to determine if there was a significant difference between variables amongst the group of patients who had a PJI within 3 months and those who did not. Correlation with pre-operative waiting time was analysed with Mann-Whitney U-test for dichotomous variables, Kruskal-Wallis test for non-dichotomous categorical variables and a Spearman Correlation Coefficient for continuous variables. The possible confounders analysed were: 1) Age; 2) Gender; 3) BMI; 4) Smoking status; 5) Type of arthroplasty performed; 6) ASA class; and 7) Duration of surgery.

The primary analysis processed pre-operative waiting time as a continuous variable. The two pre-planned dichotomized secondary analyses compared pre-operative waiting times with 24h and 48h cut-offs based on a Norwegian national quality indicator stating that hip fractures should be operated on within 48 hours, and ideally in less than 24 hours. The design of the study and all statistical analysis was performed in consultation with a statistician. Statistical analysis was done using SAS System Version 9.4.

### Results

**Fig 1:**
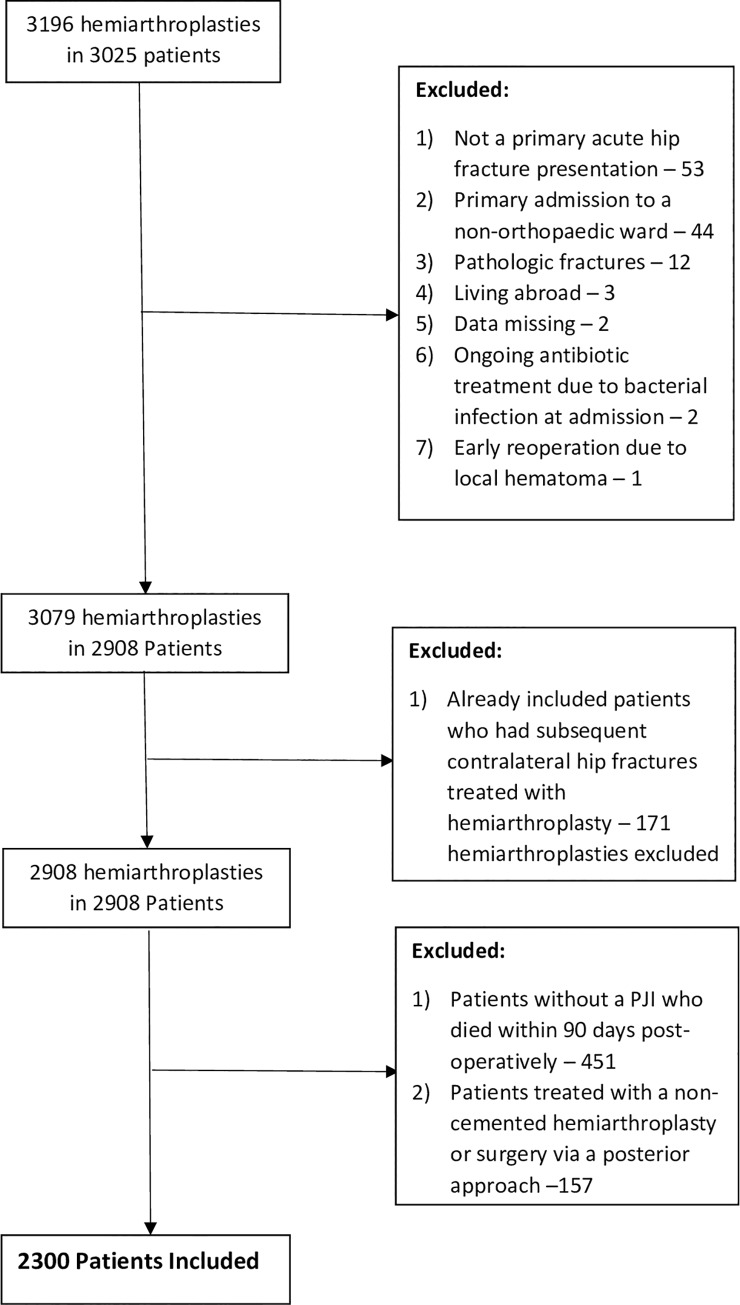
Reasons for exclusion of patients treated with a hemiarthroplasty after a hip fracture.

There was a total of 3196 patients treated with a hemiarthroplasty between 2005 and 2017 in the three centres in Norway. Of those 2300 patients were included in the final analysis. The reasons for exclusion are presented in (Fig 1). The average age of included patients was 82 years (range 48–100) and 612 (27%) were male. A total of 79 (3.4%) patients developed a PJI within 3 months of their index operation. A comparison of the demographics between patients who developed a deep PJI within 3 months, and patients who did not is presented in Table 1. The validation of the local register against the NHFR found 235 patients in the local register but not in the NHFR, and 32 in the NHFR not found in the local register. Laterality was incorrect in 13 individuals in NHFR and in 12 individuals in the local register. In the NHFR the specification of the antibiotic prophylaxis was missing in 148 individuals but not in any of those with a PJI. Based on the NHFR data 87 corrections of diagnosis codes or procedure codes were made in the local register.

**Table 1:** Demographic comparison of included patients who developed a PJI within 3 months and those who did not.

Demographic	Infection (n = 79)	No infection (n = 2221)	p value
**Age (years), mean (SD)**	82 (8.9)	82 (8.0)	
	83 (52;99)	82 (48;100)	0.44
**Gender, n (%)**			
Male	22 (28)	590 (27)	0.89
Female	57 (72)	1631 (73)	
**Pre-operative waiting time (hours), mean (min;max)**	36 (24)	34 (20)	
	28 (5.5;121)	28 (2.4;187)	0.79
**ASA Class, n (%)**			
1	0 (0.0)	13 (0.6)	
2	38 (48.1)	1037 (46.7)	0.62
3	37 (46.8)	1126 (50.7)	
4	4 (5.1)	43 (1.9)	
**Body Mass Index, mean (SD)**	25 (6.1)	24 (4.1)	
	24 (15;57)	23 (14;46)	0.55
**Smoking Status, n (%)**			
Non-Smoker	49 (63)	505 (62)	
Previous Smoker	16 (20)	148 (18)	0.72
Current Smoker	13 (17)	158 (20)	
**Duration of Surgery (min), mean (min;max)**	95 (37)	90 (25)	
	92 (45;274)	85 (30;279)	0.48

**Fig 2:**
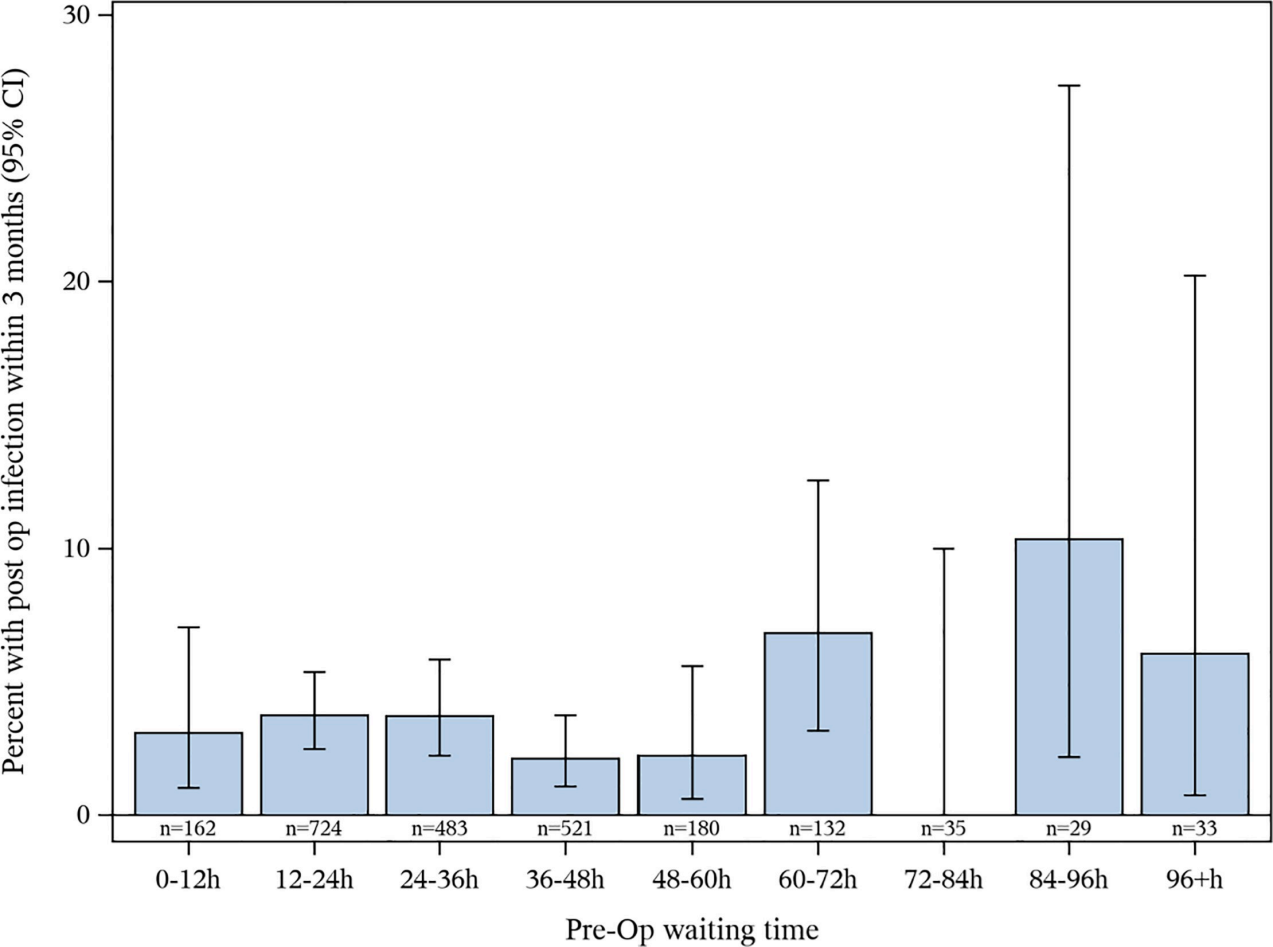
The rates of PJI within 3 months following hip hemiarthroplasty based on the time from patient presentation in the emergency department with an acute hip fracture to the time of surgery.

Mean time from presentation with hip fracture to surgery was 36 (SD 23) hours for males and 33 (SD 20)) hours for females (p = 0.04). There was a significant correlation between the time to surgery and ASA classification (r^s^ = 0.05, p = 0.002). No significant correlation was found between the time to surgery and the patients’ BMI (r_s_ = -0.01, p = 0.71), age (r_s_ = -0.03, p = 0.07), or smoking status (r_s_ = 0.07, p = 0.06). None of the demographics fulfilled the criteria to be considered a possible confounder based on differences between the infected and non-infected groups and therefore no multivariable analysis was performed (p<0.10). Fig 2 graphically shows the rates of PJI that occurred depending on time to surgery.

A univariable analysis on the effect of pre-operative waiting time on the risk of PJI, found no significant increase in risk of PJI with increased pre-operative waiting time (OR 1.06 (95% CI 0.94–1.20, p = 0.33)). Neither of the two pre-planned secondary analyses comparing pre-operative waiting time of <24 hours vs ≥24 hours (univariable analysis OR = 0.92 (95% CI 0.58–1.46, p = 0.73) and <48 hours vs ≥48 hours (univariable analysis OR = 1.39 (95% CI 0.81–2.38, p = 0.23) showed any significant difference in PJI rates.

All patients who had experienced a PJI had received prophylaxis as outlined in the methods. The first dosage of antibiotic prophylaxis was given an average of 29 (0–60) minutes prior to surgery.

## Discussion

The key finding in this study was that there was no significant increase in the rate of PJI depending on the timing of surgery based on the primary analysis. Similarly, there was no significant difference in PJI rates through comparison of either patients with pre-operative waiting times <24 hours compared with ≥24 hours or <48 hours compared with ≥48 hours.

The results of the present study differed from those of a previous study on the same topic which found that as pre-operative waiting time increased, the rate of PJI increased [15]. This may be in large part due to the fact that the study by Westberg *et al*. included 184 patients, whereas the present study had a sample size of 2300 patients [15]. Similar to our study, Zajonz *et al*. found no significant change in infection rate based on pre-operative time to HHA after femoral neck fracture, however this study also was limited by a relatively small sample size[16].

Although patients that were operated on within 48 hours from presentation did not seem to have increased risk of PJI with increased pre-operative waiting time, there are several other legitimate reasons to focus on decreasing time to operation for hip fracture patients [19]_,_[20],[21],[22]_,_[23]. However, the potential benefits of early surgery must be weighed against the need for time consuming medical optimization of the often medically complicated hip fracture patients.

The overall, rate of PJI within 3 months observed in this study was 3.4%. These PJIs occurred despite the patients received antibiotic prophylaxis prior to their index procedure. This rate of PJI is consistent with two systematic reviews which have reported an average risk of PJI after hip arthroplasty for the treatment of a hip fracture between 2–4% [10],[11]. This is a higher rate of PJI than the 0.5–2% PJI rate typically found in the primary elective hip arthroplasty population [24],[25]. It is unsurprising that hip fracture patients have higher rates of PJI than the elective hip arthroplasty population given the increased age, poor nutritional status, and large number of comorbidities of hip fracture patients [26].

The strengths of this study include its large sample size, relatively homogenous population as only cemented hemiarthroplasties performed through a lateral approach were included and the validation of the data against the NHFR. Furthermore, although no multivariable analysis was performed, we found that none of the relevant demographic factors met our pre-set criteria for being a possible confounder between patients who experience a PJI and those who did not. This study is primarily limited by its retrospective nature which introduces inherent bias into the study [27]. Additionally, the time point cut offs (24 and 48 hours) used in the pre-planned secondary analyses were chosen based on Norwegian national quality indicators [28]. The results may have differed if different time cut offs had been chosen. In this study we did not examine individual comorbidities such as diabetes and renal disease as possible confounders between the infected and non-infected groups. However, we instead used ASA as an overall comorbidity index, which has previously been methodologically validated in a previous study[29].

In conclusion, based off a large retrospective Norwegian database of hip fractures there did not appear to be a significant difference in infection rate based on pre-operative wait time to surgery.
